# A Predictive Model for Super-Response to Cardiac Resynchronization Therapy: The QQ-LAE Score

**DOI:** 10.1155/2020/3856294

**Published:** 2020-08-28

**Authors:** Xi Liu, Yiran Hu, Wei Hua, Shengwen Yang, Min Gu, Hong-Xia Niu, Li-Gang Ding, Jing Wang, Shu Zhang

**Affiliations:** ^1^State Key Laboratory of Cardiovascular Disease, Arrhythmia Center, Fuwai Hospital, National Center for Cardiovascular Diseases, Chinese Academy of Medical Sciences, Peking Union Medical College, Beijing, China; ^2^Department of Cardiology and Macrovascular Disease, Beijing Tiantan Hospital, Capital Medical University, No. 119 South Fourth Ring West Road, Beijing 100070, China

## Abstract

**Objectives:**

It is important to identify super-responders who can derive most benefits from cardiac resynchronization therapy (CRT). We aimed to establish a scoring model that can be used for predicting super-response to CRT.

**Methods:**

We retrospectively reviewed 387 CRT patients. Multivariate logistic regression analysis was performed to identify predictors for super-response (defined as an absolute increase in left ventricular ejection fraction of ≥15% at 6-month follow-up) and to create a score model. Multivariate Cox proportional-hazard regression analysis was conducted to assess associations with the long-term endpoint (defined as cardiac death/heart transplant, heart failure (HF) hospitalization, or all-cause death) across the score categories at follow-up.

**Results:**

Among 387 patients, 109 (28.2%) met super-response. In multivariable analysis, 5 independent predictors (QQ-LAE) were identified: prior no fragmented QRS (odds ratio (OR) = 3.10 (1.39, 6.94)), QRS duration ≥170 ms (OR = 2.37 (1.35, 4.12)), left bundle branch block (OR = 2.57 (1.04, 6.37)), left atrial diameter <45 mm (OR = 3.27 (1.81, 5.89)), and left ventricular end-diastolic dimension <75 mm (OR = 4.11 (1.99, 8.48)). One point was attributed to each predictor, and three score categories were identified. The proportion of super-response after 6-month CRT implantation in patients with scores 0–3, 4, and 5 was 14.6%, 40.3%, and 64.1%, respectively (*P* < 0.001). Patients with score 5 had an 88% reduction in the risk of cardiac death/heart transplant (*P*=0.042), a 71% reduction in the risk of HF hospitalization (*P*=0.048), and an 89% reduction in the risk of all-cause mortality (*P*=0.028) compared to patients with scores 0–3.

**Conclusions:**

The QQ-LAE score can be used for prediction of super-response to CRT and selection of most suitable patients in clinical practices.

## 1. Introduction

Randomized trials have demonstrated that cardiac resynchronization therapy (CRT) improves cardiac performance in patients with heart failure with reduced ejection fraction (HFrEF), and reduces mortality and heart failure (HF) hospitalization [1, 2]. In addition, several studies have indicated that CRT dramatically improves left ventricular ejection fraction (LVEF) with excellent long-term outcome in patients with HFrEF or the “super-responders” [3–5].

Although previous studies have considered varying preimplant factors for predicting a super-response to CRT, the factors they focused on are isolated and have limited benefits for clinical practices [3–6]. Currently, there remain limited studies of building a predictive model that can distinguish super-responders from eligible patients [7, 8]. Therefore, our study is aimed at identifying the predictors of super-response in patients with HFrEF who received a CRT device, and designing a simple and practical score model for super-response, considering the add-on effects of the predictors.

## 2. Methods

### 2.1. Study Population

The study population was selected from a clinical database covering 455 consecutive patients with HFrEF, who successfully underwent CRT implantation in the Arrhythmia Center of Fuwai Hospital (Beijing, China), between January 2009 and January 2017. Indication for CRT implantation was based on the guidelines [9]. Inclusion criteria of this retrospective study included the following: LVEF ≤35%, QRS width ≥130 ms, and New York Heart Association (NYHA) class II-IV, despite optimized pharmacological treatment. The study excluded patients who received CRT for pacemaker/defibrillator upgrade, died of noncardiac death causes during the 6-month follow-up period, missed the 6-month follow-up, or were lost to the follow-up in our hospital. Left bundle branch block (LBBB) was defined as QRS duration ≥120 ms, monophasic QS or rS complex in lead V_1_, and monophasic R wave with no Q waves in lead V_6_ [10]. All patients received optimal pharmacological treatment before and after implantation. This retrospective study was performed with written informed consent from all patients and approval from the ethics committee of Fuwai Hospital and in accordance with the Declaration of Helsinki.

### 2.2. Device Implantation and Program Optimization

The CRT devices were manufactured by Medtronic, St Jude Medical, Biotronik, or Boston Scientific. The coronary sinus was cannulated from left subclavian and/or cephalic entry site using a commercially available long peelable guiding sheath. The left ventricular (LV) lead was positioned in the venous system, preferably in the lateral or posterolateral vein. The right atrial and right ventricular leads were positioned conventionally in the RA appendage and the RV apex, respectively. Leads were connected to the corresponding CRT device. All procedures were performed under local anesthesia.

After the implantation, atrioventricular delay optimization was programmed individually to reach the optimal diastolic filling using the Doppler mitral inflow before discharge. V-V delay ranged from 0 to 40 ms, according to the standard of the shortest biventricular paced QRS duration.

### 2.3. Echocardiographic Evaluation

All patients underwent two-dimensional echocardiographic evaluation at baseline and 3, 6, and 12 months at our hospital. Echocardiographic parameters including left atrial diameter (LAD), left ventricular end-diastolic dimension (LVEDD), and LVEF were routinely measured (Vivid E9, GE Healthcare, and General Electric-Vingmed). LVEF was measured with the modified Simpson method. The LVEF was calculated from conventional apical two- and four-chamber images with the biplane Simpson technique. The degree of mitral regurgitation (MR) and tricuspid regurgitation (TR) was assessed semiquantitatively in four classes (none, trivial/mild, moderate, and severe).

### 2.4. Follow-Up and Definition of Super-Response

After discharge, patients were required to follow-up at our hospital typically after 3, 6, and 12 months during the first year and subsequently every 6–12 months if patients were stable. At each visit, devices were interrogated, and echocardiography and laboratory tests were also performed. All enrolled patients were followed up to July 2018.

The short-term endpoint in this study was super-response, which was defined as an absolute increase in LVEF for over 15% at 6-month follow-up. The long-term endpoint was defined as cardiac death/heart transplant, HF hospitalization, and all-cause death.

### 2.5. Data Collection and Definitions of Predictors

Baseline clinical data during hospitalization, including demographic characteristics, laboratory data, and medications, were obtained from the Fuwai Electronic Medical Record System.

We defined the history of HF as the interval between first hospitalization for HF (or first detection of LV dysfunction) and CRT implantation. Frequent premature ventricular contractions (PVCs) were defined as the presence of over 1000 PVCs recorded by 24-hour Holter monitoring [11]. Fragmented QRS with bundle branch block (BBB) morphology was defined as the presence of >2 notches (at least 1 notch more than the typical BBB) or multiple notches of the R wave, or >2 notches in the nadir of the S wave recorded in ≥2 contiguous leads in 12-lead electrocardiograms [12].

Receiver-operating characteristic (ROC) curve analysis with the Youden index was used to define the optimum cutoff values of the history of HF, QRS duration, LAD, and LVEDD before CRT implantation for super-response prediction at 6-month follow-up.

### 2.6. Statistical Analysis

Statistical analysis was performed with the SPSS 22.0 statistical software package (SPSS, Inc, IBM, Armonk, New York). Continuous variables were expressed as mean ± SD, and categorical variables as numbers and percentage. Continuous variables were transformed to binary variables using cutoffs to facilitate the formulation of an easily implemented score. Optimum cutoff values of continuous variables were identified through ROC curves with the Youden index. Group comparisons were carried out through Student's *t*-test or Mann–Whitney *U* test for continuous variables and chi-square test or Fisher's exact test for categorical variables. Differences in changes of echocardiographic parameters among the 3 score groups were analyzed through the one-way ANOVA test. The factors with *P* values <0.05 in the univariate analysis were entered into a multivariate logistic regression model with a forward stepwise method to identify the independent predictors. The accuracy of the score model was verified with the Hosmer–Lemeshow test. Multivariate Cox proportional-hazard regression analysis was used to assess for associations with the long-term endpoint across the score categories at follow-up. Kaplan–Meier analyses with a logrank test were used to assess survival across the score categories. All tests were 2-tailed, and a *P* value <0.05 was considered statistically significant.

## 3. Results

A cohort of 455 patients with HFrEF successfully underwent CRT implantation within the study period. Among them, 68 were excluded (46 underwent CRT for pacemaker/defibrillator upgrade, 2 died of noncardiac causes during the 6-months follow-up, 14 missed the 6-month follow-up, and 6 lost long-term follow-up). As a result, the remaining 387 patients were enrolled in the final analysis, and 109 (28.2%) of them met super-response.

Baseline clinical, electrocardiographic and echocardiographic characteristics of super-responders and non-/modest responders are presented in Table 1. In brief, super-responders were mostly females who had nonischemic HF etiology, shorter history of HF, LBBB, and longer QRS duration. Compared with non-/modest responders, super-responders less often had AF, frequent PVCs, fragmented QRS, and prior myocardial infarction; their LAD and LVEDD were also smaller.

**Figure 1:**
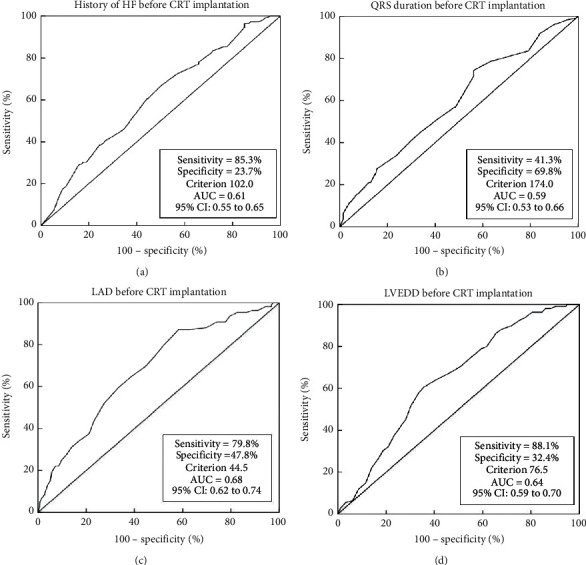
Receiver-operating characteristic curves of continuous variables predicting super-response at 6-month follow-up. (a) Receiver-operating characteristic curve of the history of HF. (b) Receiver-operating characteristic curve of QRS duration. (c) Receiver-operating characteristic curve of LAD. (d) Receiver-operating characteristic curve of LVEDD. AUC, area under the receiver-operating characteristic curve; CI, confidence interval; CRT, cardiac resynchronization therapy; HF, heart failure; LAD, left atrial diameter; LVEDD, left ventricular end-diastolic dimension.

The ROC was used to determine a cutoff of 4 significantly continuous variables as a categorical predictor for super-response. As shown in Figure 1, the area under the receiver-operating characteristic curve (AUC) for the history of HF before CRT implantation was 0.61 and a cutoff value of 102 months had a sensitivity of 85.3% and a specificity of 23.7% for super-response prediction. For the sake of clinical practicability, we finally chose 96 months (equal to eight years) as the cutoff values for the history of HF. Accordingly, AUC for QRS duration, LAD, and LVEDD before CRT implantation was 0.59, 0.68, and 0.64; the sensitivity of a 174 ms, 44.5 mm, and 76.5 mm cutoff value for QRS duration, LAD, and LVEDD was 41.3%, 79.8%, and 88.1%, respectively, and the specificity of the same sequence was 69.8%, 47.8%, and 32.4%, respectively, for super-response prediction. We finally chose 170 ms, 45 mm, and 75 mm as the cutoff values for the QRS duration, LAD, and LVEDD, respectively.

In multivariable logistic analysis, 5 independent predictors were associated with the CRT super-response at the 6-month follow-up: prior no fragmented QRS (odds ratio (OR) = 3.10 (1.39, 6.94); *P*=0.006), QRS duration ≥170 ms (OR = 2.37 (1.35, 4.12); *P*=0.003), LBBB (OR = 2.57 (1.04, 6.37); *P*=0.042), LAD <45 mm (OR = 3.27 (1.81, 5.89); *P* < 0.001), and LVEDD <75 mm (OR = 4.11 (1.99, 8.48); *P* < 0.001) (Table 2). For the scoring construct, each variable was assigned 1 point based on the *β* partial regression coefficient, a similar estimating approach for different variables. A simple acronym QQ-LAE was derived reflecting the different risk factor components of the score. The contiguous score categories were combined to form three distinct categories: low response, score 0–3 (*n* = 219); medium response, score 4 (*n* = 129); and high response, score 5 (*n* = 39). The AUC of the multivariable score was 0.71 (0.65, 0.77) (*P*=0.030), and accuracy of the score model was confirmed by the nonsignificant Hosmer–Lemeshow test (*P*=0.153). The proportion of super-response after 6-month CRT implantation in the low, medium, and high response score was 14.6%, 40.3%, and 64.1%, respectively (*P* < 0.001).

**Figure 2:**
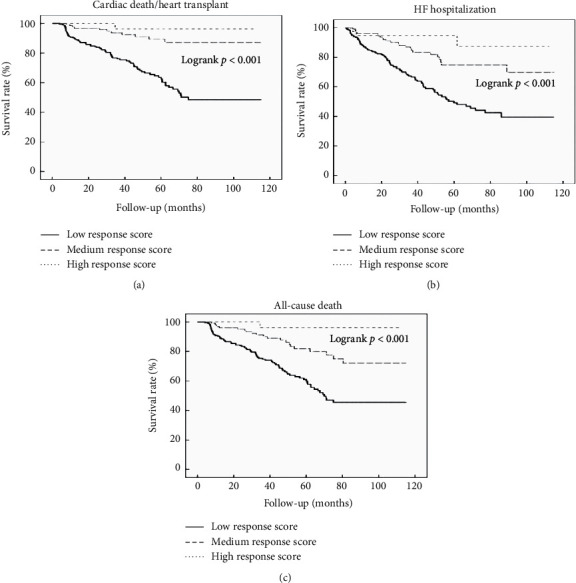
Kaplan–Meier estimates of survival rate of (a) cardiac death/heart transplant, (b) HF hospitalization, and (c) all-cause death. HF, heart failure.

In our study population, 87 patients died of cardiac causes or received heart transplant and 119 patients were hospitalized for HF during a median follow-up of 47.2 ± 26.6 months. The all-cause death occurred in 113 patients. The cardiac death/heart transplant occurred in 75 patients (34.2%) in the low response score, 11 patients (8.5%) in the medium response score, and 1 patient (2.6%) in the high response score. The HF hospitalization occurred in 92 patients (42.0%) in the low response score, 24 patients (18.6%) in the medium response score, and 3 patients (7.7%) in the high response score. The all-cause death occurred in 81 patients (36.9%) in the low response score, 21 patients (16.3%) in the medium response score, and 1 patient (2.6%) in the high response score. After adjustment for multiple comorbidities (Table 3), patients with the high response score derived significantly greater clinical benefits from CRT that include an 88% reduction in the risk of cardiac death/heart transplant (*P*=0.042), a 71% reduction in the risk of HF hospitalization (*P*=0.048), and an 89% reduction in the risk of all-cause mortality (*P*=0.028) compared to patients with the low response score. Besides, patients with the medium response score also derived significantly clinical benefits from CRT that include a 57% reduction in the risk of cardiac death/transplant (*P*=0.022) compared to patients with the low response score. The hazard ratio for HF hospitalization and all-cause death in the medium response score, as compared with the low response score, was 0.89 (95% confidence interval (CI): 0.51 to 1.55; *P*=0.679) and 0.29 (95% CI: 0.09 to 0.99; *P*=0.179).  Figure 2 shows Kaplan–Meier estimates of cardiac death/heart transplant, HF hospitalization, and all-cause death for the three groups stratified by score categories, separately.

## 4. Discussion

The major results of our study have practical significance in selection of patients for treatment with CRT and reduction of healthcare costs, especially for developing countries such as China. QQ-LAE score includes five baseline clinical and echocardiographic data: prior no fragmented QRS, QRS duration ≥170 ms, LBBB, LAD <45 mm, and LVEDD <75 mm. This simple and practical score can help identify patients with high, medium, and low response (score 5, 4, and 0–3, respectively) to CRT implantation at the 6-month follow-up. According to our results, compared to patients with the low response score, patients with medium and high response score derived significantly greater clinical benefits from CRT, including higher occurrence of super-response, lower risks of death, and HF hospitalization. Although patients with medium response score was not associated with a significantly lower risk of HF hospitalization and all-cause death, we speculated the result may be attributed to the relatively limited number of patients. To sum up, our findings imply that patients with medium or high response score before CRT implantation deserve more attention from physicians for further identification of patients with beneficial effects from CRT and better use of healthcare resources.

Previous studies focusing on creating a super-response score were limited. Goldenberg et al. identified factors associated with reverse remodeling following CRT by using data from MADIT-CRT [7]. They created a response score, including 7 factors (female sex, nonischemic origin, left bundle branch block, QRS ≥ 150 ms, prior hospitalization for HF, left ventricular end-diastolic volume ≥125 mL/m^2^, and left atrial volume <40 mL/m^2^), to identify patients who derive clinical benefit from CRT device. However, this response score is complicated and applicable only to the MADIT-CRT population who were patients with LVEF ≤30% and mild HF symptoms. Yanagisawa et al. retrospectively analyzed 80 patients who underwent CRT implantation and found 3 independent predictors for CRT super-response [8]. Their predictive model, involving a combination of the proportion of right ventricular pacing >90% before upgrading to CRT, lack of prior history of ventricular arrhythmia, and smaller LAD, can increase the possibility of predicting super-response to CRT at 6-month follow-up. Serdoz et al. used a nomogram to predict the individual probability of normal LVEF after 1 year of CRT implantation [13]. Besides, Maass et al. prospectively enrolled 240 patients who underwent CRT implantation and established a predicting model named CAVIAR score, which was consisted of four variables including age, QRS_AREA_, interventricular mechanical delay, and apical rocking. This CAVIAR response score also predicted clinical outcome assessed by heart failure hospitalizations and all-cause mortality [14]. Yet these studies have obvious limitation—the sample size is relatively small. Simple, clinically practical, and more applicable to Chinese patients who have indications for CRT implantation in real world, QQ-LAE score remains the first predictive model for super-response to CRT based on the Chinese population that incorporates 5 baseline indicators to evaluate the possibility of super-response before CRT implantation.

We identified 5 variables associated with the baseline clinical and echocardiographic indicators that made up the response score. Among the variables, wider QRS duration [3, 15], LBBB [3, 16], and LAD [6, 8] are aligned with several earlier studies; fragmented QRS is associated with cardiac fibrosis and significant intraventricular systolic dyssynchrony which may affect the response to CRT [17]. Celikyurt et al. found that the number of leads with fragmented QRS was the only predictor for response to CRT and suggested that fragmented QRS may help in the selection of CRT candidates [18]. In respect to the last variable LVEDD, Díaz-Infante et al. showed that LVEDD ≥75 mm was one of the predictors for lack of response to CRT [19]. They speculated that dilated LVEDD may be a signal of more advanced cardiac disease and poorer prognosis. In a recent study by Tian et al., a smaller LVEDD was observed in patients with CRT super-response [20]. We also observed that prior no fragmented QRS and smaller LVEDD are independent predictors of super-response to CRT as shown in Table 2. According to our data, prior no fragmented QRS and LVEDD <75 mm provides additional information on response to CRT. Other common predictive factors for super-response to CRT, such as female sex [3, 4] and a nonischemic origin of cardiomyopathy [3, 15], and multivariate logistic analysis did not show significant differences in our study. Besides, male sex and nonischemic cardiomyopathy were most common in Asian population according to previous studies [20, 21]. We showed similar results that proportions of male sex and nonischemic cardiomyopathy were 64.6% and 81.1%, respectively. Thus, it is possible that the sample size may be one potential explanation for these results. However, our baseline results showed that female sex and nonischemic origin of cardiomyopathy were more common in super-responders than non-/modest responders. Besides, Maass et al. and Van Stipdonk et al. demonstrated that vectorcardiographic QRS area performs better than conventional electrocardiography to predict super-response after CRT. The strong association of vectorcardiographic derived QRS area and CRT response may be explained by the fact that it shows the extent of unopposed electrical forces generated within the heart during ventricular depolarization, representing the direction as well as the delay of electrical activation [14, 22].

It should be noted that there is no score or model that enables predictions of 100 percent probability of individual super-response occurrence to CRT. We created QQ-LAE score to assist clinical physicians to evaluate the individual possibility of super-response before CRT implantation. For example, a symptomatic patient with HF in sinus rhythm with a QRS duration ≥150 ms but <170 ms and LBBB and with LVEF ≤35% would have class I indication for CRT implantation based on the latest guidelines [9]. According to QQ-LAE score, the patients would not be considered as the high response score (QRS duration is ineligible); this information would help physicians to predict relatively accurate occurrence of super-response to CRT rather than roll out the option for CRT implantation. If the patient only has one ineligible QRS duration (QRS duration <170 ms), the probability of super-response occurrence is nearly 50% according to the score. The probability will further decrease if an additional fragmented QRS is combined or if LVEDD is expanded (≥75 mm)—both cases will lead to lower score of the patient. Hence, QQ-LAE score provides a simple approach to response assessment in candidates, who may derive most benefits from CRT implantation.

## 5. Conclusions

The QQ-LAE score is derived from five common baseline clinical and echocardiographic indicators that have an add-on predictive effect on super-response to CRT. While further validation in larger cohorts will be needed, this simple score model can be used for prediction of super-response to CRT and selection of most suitable patients in clinical practices.

## Figures and Tables

**Table 1:** Baseline characteristics according to CRT response.

Clinical parameters	Non-/modest responders (*n* = 278)	Super-responders (*n* = 109)	*P* value
Age, years	59.0 ± 11.2	58.1 ± 9.9	0.463
Age ≥ 65, *n* (%)	87 (31.3)	28 (25.7)	0.278
Women, *n* (%)	88 (31.7)	49 (42.2)	0.014
BMI, kg/m^2^	24.5 ± 4.3	24.3 ± 4.2	0.781
History of HF, months	66.1 ± 58.7	44.9 ± 44.5	<0.001
History of HF ≤ 96 months, *n* (%)	199 (71.6)	91 (83.5)	0.015
NYHA functional class, *n* (%)			0.357
II	49 (17.6)	24 (22.0)	
III	185 (66.5)	73 (66.9)	
IV	44 (15.8)	12 (11.0)	
Nonischemic etiology, *n* (%)	218 (78.4)	96 (88.1)	0.029
AF, *n* (%)	27 (9.7)	4 (3.7)	0.049
Frequent PVCs, *n* (%)	155 (55.8)	44 (40.4)	0.006
Fragmented QRS, *n* (%)	67 (24.1)	12 (11.0)	0.004
LBBB, *n* (%)	219 (78.8)	102 (93.6)	<0.001
QRS duration, ms	163.8 ± 17.4	169.9 ± 18.7	0.002
QRS duration ≥ 170 ms, *n* (%)	84 (30.2)	45 (41.3)	0.038
Hypertension, *n* (%)	87 (31.3)	38 (34.9)	0.500
Diabetes, *n* (%)	74 (26.6)	21 (19.3)	0.131
Coronary heart disease, *n* (%)	76 (27.3)	27 (24.8)	0.607
Prior MI, *n* (%)	42 (15.1)	7 (6.4)	0.021
LVEF, %	27.3 ± 5.3	27.5 ± 4.7	0.763
LVEDD, mm	72.0 ± 9.7	67.2 ± 7.5	<0.001
LVEDD <75 mm, *n* (%)	182 (65.5)	94 (86.2)	<0.001
LAD, mm	43.7 ± 8.1	38.5 ± 8.1	<0.001
LAD <45 mm, *n* (%)	145 (52.2)	87 (79.8)	<0.001
MR grade, *n* (%)			0.252
None	34 (12.2)	16 (14.6)	
Trival/mild	92 (33.1)	44 (40.4)	
Moderate	102 (36.7)	37 (33.9)	
Severe	50 (17.9)	12 (11.0)	
TR grade, *n* (%)			0.377
None	23 (8.3)	5 (4.6)	
Trival/mild	162 (58.3)	62 (56.9)	
Moderate	68 (24.5)	34 (31.2)	
Severe	25 (8.9)	8 (7.3)	
Beta-blocker, *n* (%)	265 (95.3)	104 (95.4)	0.970
ACEI/ARB, *n* (%)	255 (91.7)	102 (93.6)	0.540
Aldosterone antagonists, *n* (%)	237 (85.3)	97 (88.9)	0.336

Data are presented as mean ± SD or percentage. ACEI, angiotensin-converting enzyme inhibitor; AF, atrial fibrillation; ARB, angiotensin receptor blockers; BMI, body mass index; CRT, cardiac resynchronization therapy; HF, heart failure; LAD, left atrial diameter; LBBB, left bundle branch block; LVEDD, left ventricular end-diastolic diameter; LVEF, left ventricular ejection fraction; MI, myocardial infarction; MR, mitral regurgitation; NYHA, New York heart association; PVCs, premature ventricular contractions; TR, tricuspid regurgitation.

**Table 2:** Predictors of CRT super-response, and uni- and multivariate logistic regression models.

	Univariate		Multivariate		
	OR (95% CI)	*P*-value	*β*	OR (95% CI)	*P* value
Sex (female)	1.76 (1.12–2.78)	0.014			
History of HF ≤ 96 months	2.01 (1.14–3.55)	0.016			
Nonischemic etiology	2.03 (1.07–3.88)	0.031			
Prior no AF	2.82 (0.96–8.27)	0.058			
Prior no frequent PVCs	1.86 (1.19–2.92)	0.007			
Prior no fragmented QRS	2.57 (1.33–4.97)	0.005	1.131	3.10 (1.39–6.94)	0.006
LBBB	3.93 (1.73–8.89)	0.001	0.933	2.57 (1.04–6.37)	0.042
QRS duration ≥170 ms	1.97 (1.22–3.18)	0.006	0.862	2.37 (1.35–4.12)	0.003
Prior no MI	2.59 (1.13–5.97)	0.025			
LVEDD <75 mm	3.31 (1.82–6.01)	<0.001	1.407	4.11 (1.99–8.48)	<0.001
LAD <45 mm	3.63 (2.15–6.12)	<0.001	1.179	3.27 (1.81–5.89)	<0.001

CI, confidence interval; OR, odds ratio; *β*, *β* partial regression coefficient. AF, atrial fibrillation; CRT, cardiac resynchronization therapy; HF, heart failure; LAD, left atrial diameter; LBBB, left bundle branch block; LVEDD, left ventricular end-diastolic diameter; MI, myocardial infarction; PVCs, premature ventricular contractions.

**Table 3:** Cox proportional-hazard regression analysis of score categories of long-term endpoint.

Endpoint and score	No. of patients	No. of patients with events (%)	Adjusted HR	95% CI	*P* value
Cardiac death/heart transplant					
Low response score	219	75 (34.2)	1.00		
Medium response score	129	11 (8.5)	0.43	0.21 to 0.89	0.022
High response score	39	1 (2.6)	0.12	0.02 to 0.93	0.042

HF hospitalization					
Low response score	219	92 (42.0)	1.00		
Medium response score	129	24 (18.6)	0.89	0.51 to 1.55	0.679
High response score	39	3 (7.7)	0.29	0.09 to 0.99	0.048

All-cause death					
Low response score	219	81 (36.9)	1.00		
Medium response score	129	21 (16.3)	0.29	0.09 to 0.99	0.179
High response score	39	1 (2.6)	0.11	0.01 to 0.79	0.028

CI, confidence interval; HR, hazard ratio; 95%; HF, heart failure.

## Data Availability

Data are available upon contacting the first author.

## Additional Points

This study has a few limitations. Firstly, this was a retrospective and single-center study with a limited number of patients. Further prospective multicenter studies with large sample size are required to validate and examine this predictive score for super-response to CRT. Secondly, LVEF was defined as the indicator of super-response in this study while previous studies used left ventricular end systolic volume as the indicator. The definition of LBBB was based on ESC criteria in this study while AHA, Strauss, and MADIT criteria were also used in previous studies [23]. So, the differences in classifying super-response and LBBB were present, which were inevitable biases in this study. Besides, the data of vectorcardiographic QRS area, a good predictor of super-response in CRT, were not available in this study. Finally, a 6-month follow-up may be too short to correctly identify super-responders. LVEF in a small number of patients could be observed a still improvement after a longer follow-up, called delayed response [24].
